# Genotypic analysis of a large cohort of patients with suspected atypical hemolytic uremic syndrome

**DOI:** 10.1007/s00109-023-02341-4

**Published:** 2023-07-19

**Authors:** Dervla M. Connaughton, Pratibha Bhai, Paul Isenring, Mohammed Mahdi, Bekim Sadikovic, Laila C. Schenkel

**Affiliations:** 1 Schulich School of Medicine & Dentistry, University of Western, London, ON Canada; 2 Molecular Genetics Laboratory, Molecular Diagnostics Division, London Health Sciences Centre (LHSC), London, ON Canada; 3 Faculty of Medicine, Université Laval, Quebec City, QC Canada; 4 Alexion Pharmaceuticals, Inc, Boston, MA USA; 5 Department of Medicine, Division of Nephrology, London Health Sciences Centre, 339 Windermere Road, London, ON Canada; 6 Pathology and Laboratory Medicine, Western University, London, ON Canada

**Keywords:** Atypical hemolytic uremic syndrome, aHUS, Complement, Coagulation, Next-generation sequencing, Thrombotic microangiopathy

## Abstract

**Abstract:**

Atypical hemolytic uremic syndrome (aHUS) is characterized by microangiopathic hemolytic anemia, thrombocytopenia, and renal impairment. Complement and coagulation gene variants have been associated with aHUS susceptibility. We assessed the diagnostic yield of a next-generation sequencing (NGS) panel in a large cohort of Canadian patients with suspected aHUS. Molecular testing was performed on peripheral blood DNA samples from 167 patients, collected between May 2019 and December 2021, using a clinically validated NGS pipeline. Coding exons with 20 base pairs of flanking intronic regions for 21 aHUS-associated or candidate genes were enriched using a custom hybridization protocol. All sequence and copy number variants were assessed and classified following American College of Medical Genetics guidelines. Molecular diagnostic results were reported for four variants in three individuals (1.8%). Twenty-seven variants of unknown significance were identified in 25 (15%) patients, and 34 unique variants in candidate genes were identified in 28 individuals. An illustrative patient case describing two genetic alterations in complement genes is presented, highlighting that variable expressivity and incomplete penetrance must be considered when interpreting genetic data in patients with complement-mediated disease, alongside the potential additive effects of genetic variants on aHUS pathophysiology. In this cohort of patients with suspected aHUS, using clinical pipelines for genetic testing and variant classification, pathogenic/likely pathogenic variants occurred in a very small percentage of patients. Our results highlight the ongoing challenges in variant classification following NGS panel testing in patients with suspected aHUS, alongside the need for clear testing guidance in the clinical setting.

**Key messages:**

• Clinical molecular testing for disease associated genes in aHUS is challenging.

• Challenges include patient selection criteria, test validation, and interpretation.

• Most variants were of uncertain significance (31.7% of patients; VUS + candidates).

• Their clinical significance may be elucidated as more evidence becomes available.

• Low molecular diagnostic rate (1.8%), perhaps due to strict classification criteria.

• Case study identified two likely pathogenic variants; one each in *MCP/CD46* and *CFI*.

**Supplementary Information:**

The online version contains supplementary material available at 10.1007/s00109-023-02341-4.

## Introduction

Hemolytic uremic syndrome (HUS) is a condition which presents as thrombotic microangiopathy (TMA) and is characterized by microangiopathic hemolytic anemia (MAHA), thrombocytopenia, and renal impairment [1, 2]. The majority of HUS cases are caused by Shiga toxin-producing *E. coli* infections [1]; other secondary causes include drugs, autoimmune diseases, and cobalamin deficiency [3, 4]. A small proportion of cases present with a rare, familial, or sporadic form of HUS, known as atypical HUS (aHUS) [1, 2]. aHUS diagnosis is complex and involves eliminating other common causes of TMA, testing for serum complement levels and identifying genetic variants in aHUS-associated genes.aHUS is typically caused by overactivation of the alternative complement system, leading to endothelial damage [5], inflammation, and activation of the coagulation cascade [6]. Variants in genes encoding complement regulatory proteins, such as complement factor H (CFH), complement factor I (CFI), membrane cofactor protein (MCP, also known as CD46), complement component 3 (C3), complement factor B (CFB) [7], or the presence of anti-CFH antibodies [2], have all been linked with aHUS. Variants in genes encoding coagulation factors have also been linked with aHUS. These genes include *diacylglycerol kinase epsilon* (*DGKE*), *thrombomodulin* (*THBD*), *factor XII* (*F12*), *von Willebrand factor* (*VWF*), and *plasminogen* (*PLG*) [1, 6, 8–10]. Variants in *PLG* were identified as the second most common deleterious variants in aHUS after those in *CFH*, suggesting that this gene, and the coagulation pathway, may contribute to aHUS development [9]. However, aHUS manifestation may also be triggered by other genetic or environmental events [3]. Initial studies identified pathogenic variant/s in complement genes in 50–60% of patients [8, 9, 11]; however, these studies used less stringent criteria for variant classification than the specifications of the American College of Medical Genetics (ACMG) guidelines for constitutional clinical testing [12].

Prior to the availability of targeted therapies, first-line treatment of aHUS consisted of plasma therapy, which was associated with poor prognosis; > 50% of patients reached end-stage kidney disease (ESKD) or death at 3 years [1, 8, 13]. Eculizumab, the first complement C5 inhibitor, was approved in 2011 for the treatment of aHUS and has been shown to inhibit complement-mediated TMA and significantly improve long-term hematologic and renal function in patients with aHUS [14, 15]. The C5 inhibitor ravulizumab, designed to have an extended half-life, was approved by the Food and Drug Administration for the same indication in 2019 [16] and for paroxysmal nocturnal hemoglobinuria by Health Canada in 2019 [15]. As of writing, it is under review for use in aHUS by the Canadian Agency for Drugs and Technologies in Health.

Screening for genetic alterations in complement and coagulation genes can help confirm aHUS diagnoses and inform treatment options. Hence, clinical practice guidelines recommend genetic testing for all patients presenting with aHUS to guide prognosis and treatment [17]. Next-generation sequencing (NGS) technology allows for high-throughput sequencing of multiple genes and has been used to detect genetic variants in patients with aHUS [6, 11]. However, due to the rarity of aHUS and limited numbers of case reports and functional studies, identified variants are often of unknown significance, with uncertain biologic or clinical relevance [3, 18]. Genetic evaluation of aHUS is also challenging due to the incomplete penetrance and variable expressivity of aHUS-associated genes, limited information about the nature of gene variants with unknown association with aHUS, and the presence of additional triggers that can cause disease manifestation. At the London Health Sciences Centre (LHSC), we implemented an NGS panel for clinical genetic testing of patients with suspected aHUS. The aim of this study was to assess the diagnostic yield of this proposed NGS panel test in the screening of patients with an aHUS indication and to explore the nature of genetic variants, including single nucleotide variants and copy number variants, using a previously designed NGS pipeline.

## Methods

### Patient disposition and sampling

A total of 167 patients with confirmed or suspected aHUS diagnoses were referred from institutions across Canada between May 2019 and December 2021. The major indications for testing were histological evidence of TMA and/or MAHA. While most patients did not have a confirmed aHUS diagnosis and other TMA causes were not necessarily ruled out, aHUS is typically diagnosed in Canadian hospitals on the basis of mechanical hemolytic anemia with thrombocytopenia in the absence of TTP or HUS criteria. Peripheral blood samples were collected at referring centers and sent to the Molecular Diagnostic Laboratory at LHSC, where NGS was performed on extracted peripheral blood DNA. Further information was collected from the internal clinical laboratory quality assurance and quality control database.

### Genetic analysis

Genetic testing was performed using a previously validated and extensively described NGS pipeline [19, 20]. All coding exons along with 20 base pairs (bp) of flanking intronic regions were enriched for 21 aHUS-associated or candidate genes (two isoforms of three genes tested, Supplementary Table 1) using a custom LHSC-targeted hybridization protocol (Roche NimbleGen, Madison, Wisconsin, USA). This panel targets a comprehensive list of genes including the confirmed aHUS-associated genes: *C3*, *CD46*, *CFB*, *CFH*, *CFHR1–5*, *CFI*, *DGKE*, *INF2*, *MMACHC*, and *MMADHC*. Candidate genes, based on literature reports available at the time of panel generation, included *C9*, *F12*, *PLG*, *ST3GAL1*, *THBD*, and *VWF*. Libraries were sequenced using the MiSeq V2 reagent kit to generate two 150 bp paired-end reads using the MiSeq fastq generation mode (Illumina, San Diego, CA).

Sequence analysis for variant identification was assessed and interpreted using clinically validated algorithms and commercial software (NextGene software V.2.4.2 [SoftGenetics]; Geneticist Assistant; Mutation Surveyor; and Alamut Visual). Copy number variation (CNV) analysis was performed according to a previously developed protocol based on normalized read depth [20]. Thresholds were set at mean read depth coverage of > 300 × , with a minimum 100 × coverage at a single nucleotide resolution; this assay has been validated at a level of sensitivity and specificity of combined Sanger sequencing and Multiplex Ligation-dependent Probe Amplification (MLPA) copy number analysis (> 99%) [19, 20].

All genetic variants identified by NGS were classified for pathogenicity into one of five categories according to the ACMG guidelines: ACMG category 1 (pathogenic); 2 (likely pathogenic); 3 (variant of unknown significance [VUS]); 4 (likely benign); and 5 (benign) [12]. Only ACMG categories 1, 2, and 3 are reported in this study. Complex rearrangements or variants detected at low variant frequency were confirmed using Sanger sequencing, Multiplex Ligation-dependent Probe Amplification (MLPA), or other assays.

### Case study

Clinico-biological data for one patient who underwent genetic analysis are presented to illustrate the challenges with interpreting genetic abnormalities in aHUS.

## Results

### Quality assessments

Genetic testing was distributed across 55 NGS runs; a total of 21 genes (with two isoforms of three genes) and 271 coverage regions formed the target aHUS panel tested in each run. The average and minimum coverages for each gene are described in Supplementary Table 1. All coverage regions passed the predefined thresholds.

### Demographic information

Of the 167 individuals, 89 were female, 77 were male, and one was non-identified. The mean (standard deviation) age was 43.3 (18) years, and ~ 96% of the tested individuals were ≥ 18 years old.

### Variant analysis

Of the 167 individuals tested, 28 (17%) showed at least one reportable variant (VUS or pathogenic/likely pathogenic (P/LP)), in an aHUS-associated gene. A molecular diagnostic result, including patients with at least one P/LP variant in an autosomal dominant gene or two variants in an autosomal recessive gene, was reported in three individuals (1.8%), a rate consistent with the expected prevalence of genetic aHUS in all HUS cases. Four P/LP variants were identified in these three patients, including the genes *MCP/CD46*, *CFH*, *CFI*, and *DGKE* (Table 1). One patient presented with two LP variants in the *MCP/CD46* and *CFI* genes (see case report), while an isolated *CFH* heterozygous LP variant was observed in a second patient. The third patient was homozygous for a pathogenic variant in the *DGKE* gene, associated with autosomal recessive aHUS susceptibility.

**Table 1:** List of P/LP variants, VUS, and candidate gene variants identified in individuals with a clinical or suspected diagnosis of aHUS

Gene	Nucleotide change	Amino acid change	No. of individuals	Inheritance for familial aHUS
Pathogenic/likely pathogenic (P/LP) variants				
***CD46***	**191G > T**	**Cys64Phe**	1*	**AD**
***CFH***	**3643C > G**	**Arg1215Gly**	**1**	**AD**
***CFI***	**1071T > G**	**Ile357Met**	1*	**AD**
***DGKE***	**1068_1071delTAAA**	Asn356Lysfs*6	**1 (homozygous)**	**AR**
Variants of unknown significance (VUS)				
*C3*	3655C > T	Arg1219Cys	1	AD
	1345G > A	Val449Met	1	
	4535G > A	Arg1512His	1	
*CFB*	1019A > T	Asn340Ile	1	AD
	724A > C	Ile242Leu	1	
*CFH*	1949G > T	Gly650Val	1	AD
	2675C > T	Ala892Val	1	
*CFHR1*	19delG	Val7*	1	Risk factor
	850G > A	Ala284Thr	1	
*CFHR2*	631C > T	Gln211*	1	Risk factor
	760C > T	Arg254*	1	
	334_337delATTA	Ile112Phefs*18	1	
*CFHR3*	839_840del	Ile280Lysfs*7	1	Risk factor
*CFHR4*	1607 T > G	Ile536Arg	1	Risk factor
*CFHR5*	1106 T > G	Val369Gly	1	Risk factor
*CFI*	377 T > C	Ile126Thr	1	AD
	355G > A	Gly119Arg	1	
	550G > A	Val184Met	1	
	1246A > C	Ile416Leu	1	
	1289 T > C	Ile430Thr	1	
*DGKE*	1679A > G	Gln560Arg	2	AR
*INF2*	2509C > T	Arg837Cys	1	AD (nephrotic syndrome)
	2654C > G	Ala885Gly	1	
	638C > T	Ala213Val	1	
*MMACHC*	683C > T	Ala228Val	1	AR
	8C > G	Pro3Arg	1	
	848G > C	*283Ser	1	
Candidate genetic variants				
*C9*	580C > T	Arg194*	1	
	346C > T	Arg116*	1	
	1111 + 3A > G		1	
	1645 + 2 T > G		1	
	376G > A	Gly126Arg	1	
	499C > T	Pro167Ser	1	
	1014 T > A	Phe338Leu	3**	
*F12*	1570G > T	Val524Leu	1	
	55 T > G	Ser19Ala	1	
*PLG*	1157G > A	Arg386Gln	1	
	1469G > A	Arg490Gln	1	
	758G > A	Arg253His	1	
	782G > A	Arg261His	1	
*ST3GAL1*	166C > T	His56Tyr	1	AR
*THBD*	696G > T	Gln232His	1	
	1057C > A	Pro353Thr	1	
*VWF*	409G > T	Val137Leu	1	
	4103 T > C	Ile1368Thr	1	
	4751A > G	Tyr1584Cys	1	
	5278G > A	Val1760Ile	2**	
	55 + 8C > A		2**	
	2561G > A	Arg854Gln	2**	
	5761C > T	Arg1921Trp	1	
	5793G > C	Gln1931His	1	
	7150C > T	Arg2384Trp	1	
	74G > T	Gly25Val	1	
	7604G > A	Arg2535Gln	1	
	7940C > T	Thr2647Met	1	
	7624A > G	Ile2542Val	1	
	7988G > C	Arg2663Pro	1	
	8296G > A	Asp2766Asn	1	
	8366C > A	Thr2789Asn	1	
	8378 T > C	Val2793Ala	2**	
	974G > T	Cys325Phe	1	

aHUS molecularly confirmed cases are in bold; all variants presented as heterozygous unless otherwise specified

*ACMG* American College of Medical Genetics, *C3* complement component 3, *C9* complement component 9, *CD46* cluster of differentiation 46, *CFB* complement factor B, *CFH* complement factor H, *CFHR1* complement factor H-related 1, *CFI* complement factor I, *DGKE* diacylglycerol kinase epsilon, *INF2* inverted formin-2, *MMACHC* methylmalonic aciduria and homocystinuria type C protein, *PLG* plasminogen, *P/LP* pathogenic/likely pathogenic, *THBD* thrombomodulin, *VWF* von Willebrand factor, *VUS* variant of unknown significance

*Presented as a case study

**Unrelated individuals

**Fig. 1:**
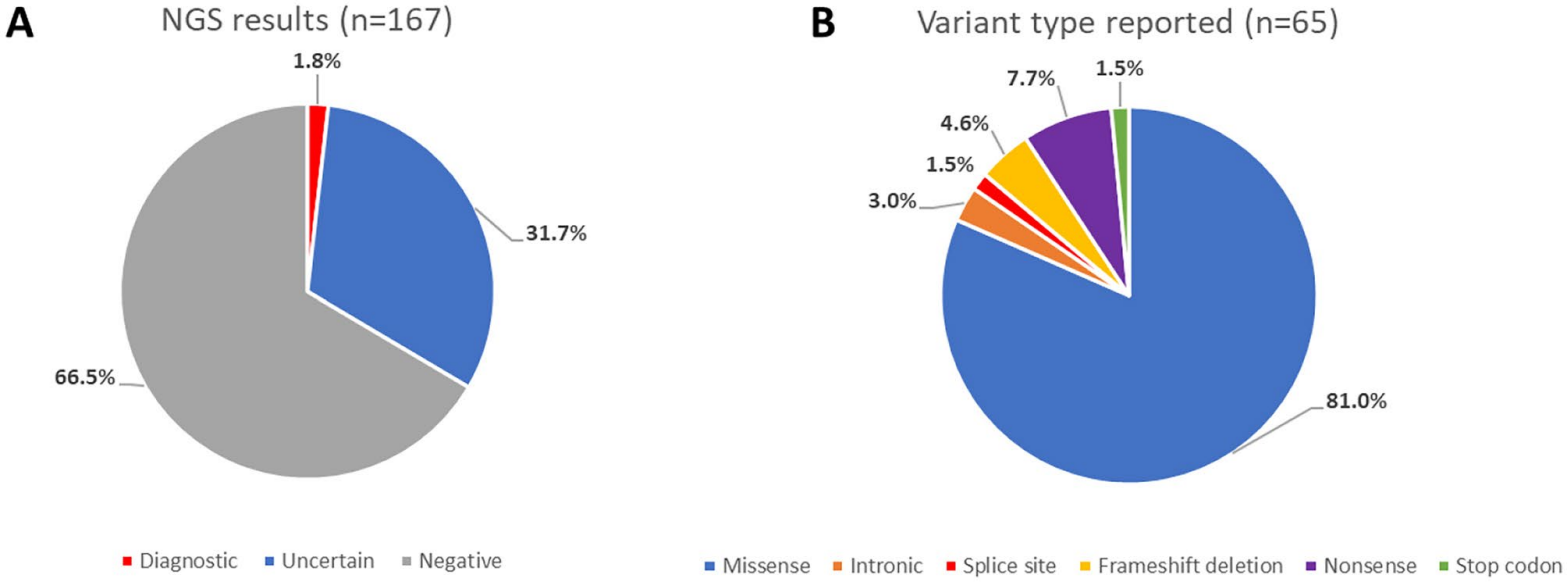
Summary of NGS aHUS panel testing. **A** Molecular testing results of 167 patients referred for aHUS testing. Diagnostic (red): patients with at least one pathogenic (P) or likely pathogenic (LP) variant in an autosomal dominant gene or 2 P or LP variants in an autosomal recessive gene for aHUS. Uncertain (blue): patients with one or more variants of unknown clinical significance reported or variants in candidate genes. Negative (grey): patients with no variants reported. **B** Distribution of variant types reported (total = 65 variants)

Twenty-five individuals (15%) presented with 27 VUS in an aHUS-associated gene, while 28 individuals (16.8%) presented with 34 unique variants in candidate variants (Table 1); the clinical significance of these candidate variants in the context of aHUS is not well established. Overall, 31.7% of patients had variants with an uncertain significance (VUS + candidate genes; Fig. 1A). No clinically significant or candidate variants were observed in 111 individuals (66.5%).

The evidence used for classification of variants according to ACMG guidelines is shown in Table 2. Most variants were single nucleotide variants, including missense and nonsense variants, with a small proportion of frameshift deletions and intronic variants (Fig. 1B). A lack of literature, clinical reports, and/or functional studies makes it difficult to interpret most variants, especially missense variants, resulting in an enrichment of variants classified as of unknown significance.

**Table 2:** Summary of evidence for classification of variants

Gene	Nucleotide change	Amino acid change	In silico prediction	Max. population frequency (gnomAD)	ClinVar	Literature PubMed IDs (PMIDs)
Pathogenic/likely pathogenic (P/LP) variants						
***CD46***	191G > T	Cys64Phe	4/4 damaging	NR	NR	19376828 24005975 26054645
***CFH***	3643C > G	Arg1215Gly	3/4 damaging	NR	P	646435 9551389
***CFI***	1071 T > G	Ile357Met	3/4 damaging	0.00360%		29292855 31517156 28942469 32510551
***DGKE***	1068_1071del	Asn356Lysfs*6		0.00081%	P	33841858
Variants of unknown significance (VUS)						
***C3***	3655C > T	Arg1219Cys	3/4 damaging	0.00420%	NR	NR
	1345G > A	Val449Met	4/4 benign	0.00080%	NR	NR
	4535G > A	Arg1512His	2/4 damaging	0.01000%	LB/VUS	NR
***CFB***	1019A > T	Asn340Ile	3/4 benign	NR	NR	NR
	724A > C	Ile242Leu	4/4 benign	0.11%	LB/VUS	NR
***CFH***	1949G > T	Gly650Val	4/4 benign	0.02%	LB/VUS	29888403 17018561
	2675C > T	Ala892Val	4/4 benign	0.01%	NR	NR
***CFHR1***	19delG	Val7*		0.35% (AFR: 2.61%)	NR	
	850G > A	Ala284Thr	3/4 benign	NR	NR	NR
***CFHR2***	631C > T	Gln211*		0.01%	NR	
	760C > T	Arg254*		0.08%	NR	
	334_337del	Ile112Phefs*18		0.14% (EAS: 1.86%)	B	
***CFHR3***	839_840del	Ile280Lysfs*7		0.11%	VUS	29924949 19745068
***CFHR4***	1607 T > G	Ile536Arg	4/4 benign	0.01%	NR	NR
***CFHR5***	1106 T > G	Val369Gly	3/4 benign	NR	NR	NR
***CFI***	377 T > C	Ile126Thr	3/4 benign	0.002%	NR	NR
	355G > A	Gly119Arg	3/4 damaging	0.04%	LB/LP, VUS	23685748 20513133 26691988
	550G > A	Val184Met	2/2 damaging	NR	NR	NR
	1246A > C	Ile416Leu	2/2 damaging	0.12% (AFR: 1.18%)	B/LB	29292855 24036952 23307876 20016463
	1289 T > C	Ile430Thr	3/4 damaging	0.0004%	NR	NR
***DGKE***	1679A > G	Gln560Arg	4/4 benign	0.18%	LB/VUS	28720077
***INF2***	2509C > T	Arg837Cys	2/3 damaging	0.024%	VUS	NR
	2654C > G	Ala885Gly	2/3 benign	NR	NR	NR
	638C > T	Ala213Val	2/3 benign	0.0018%	VUS	NR
***MMACHC***	683C > T	Ala228Val	4/4 damaging	0.0086% (EAS: 0.12%)	VUS	30157807
	8C > G	Pro3Arg	2/3 benign	0.012%	NR	NR
	848G > C	*283Serext*14		0.024%	VUS	NR
Candidate genetic variants						
***C9***	580C > T	Arg194*		0.008%	LP	
	346C > T	Arg116*		0.079% (EAS: 1.01%)	P	9703418 11359403 9570574 22197721
	1111 + 3A > G		Mild effect on splicing	NR	NR	NR
	1645 + 2 T > G		Abolish splice site	NR	NR	NR
	376G > A	Gly126Arg	2/3 damaging	0.02%	VUS	24036952
	499C > T	Pro167Ser	2/3 damaging	0.52%	LB/risk factor	24036952 29767720
	1014 T > A	Phe338Leu	2/3 benign	0.11%	LB	NR
***F12***	1570G > T	Val524Leu	3/3 benign	0.005%	NR	NR
	55 T > G	Ser19Ala	4/4 benign	NR	NR	NR
***PLG***	1157G > A	Arg386Gln	4/4 damaging	0.0028%	NR	NR
	1469G > A	Arg490Gln	4/4 damaging	0.12%	VUS	NR
	758G > A	Arg253His	3/3 damaging	0.082% (ASJ: 0.30%)	LB	NR
	782G > A	Arg261His	3/3 damaging	0.25% (FIN: 0.50%)	B/VUS	27194806
***ST3GAL1***	166C > T	His56Tyr	4/4 benign	0.00%	NR	NR
***THBD***	696G > T	Gln232His	2/3 damaging	NR	NR	NR
	1057C > A	Pro353Thr	3/3 benign	0.0018%	NR	NR
***VWF***	2561G > A	Arg854Gln	4/4 damaging	0.35%	P	20586924 15461624 1906877
	409G > T	Val137Leu	3/3 benign	0.033% (AFR: 0.34%)	NR	NR
	4103 T > C	Ile1368Thr	3/3 damaging	0.0028%	VUS	NR
	4751A > G	Tyr1584Cys	2/3 benign	0.26%	LP/VUS	15755288 17190853 12649144 16985174
	5278G > A	Val1760Ile	3/3 benign	0.076% (NFE: 0.14%)	LP/VUS	17190853 29924855 28971901
	55 + 8C > A		Mild effect on splicing	0.19% (AFR: 1.71%)	VUS	
	5761C > T	Arg1921Trp	3/3 damaging	0.0078%	NR	NR
	5793G > C	Gln1931His	2/3 benign	0.032% (SAS: 0.26%)	NR	24675615
	7150C > T	Arg2384Trp	4/4 damaging	0.028% (AFR: 0.22%)	VUS	22315491
	74G > T	Gly25Val	2/4 damaging	NR	NR	NR
	7604G > A	Arg2535Gln	3/4 benign	0.0095%	NR	24675615 29984440
	7940C > T	Thr2647Met	3/4 benign	0.38%	VUS	17190853 17596142 31064749
	7624A > G	Ile2542Val	3/3 benign	0.06% (SAS: 0.48%)	NR	NR
	7988G > C	Arg2663Pro	3/4 benign	0.14%	LP/VUS	16985174 27320760
	8296G > A	Asp2766Asn	4/4 benign	0.00071%	NR	NR
	8366C > A	Thr2789Asn	3/4 benign	0.0050%	VUS	NR
	8378 T > C	Val2793Ala	4/4 benign	0.060% (AFR: 0.58%)	VUS	22197721
	974G > T	Cys325Phe	4/4 damaging	0.038% (AFR: 0.40%)	VUS	26986123

*AFR* African, *ASJ* Ashkenazi Jewish, *B* benign, *C3* complement component 3, *C9* complement component 9, *CD46* cluster of differentiation 46, *CFB* complement factor B, *CFH* complement factor H, *CFHR* complement factor H-related, *CFI* complement factor I, *DGKE* diacylglycerol kinase epsilon, *EAS* East Asian, *F12* coagulation factor XII, *FIN* Finnish, *INF2* inverted formin-2, *LB* likely benign, *LP* likely pathogenic, *MMACHC* metabolism of cobalamin-associated C, *NFE* non-Finnish European, *NR* not reported, *P* pathogenic, *PLG* plasminogen, *SAS* South Asian, *ST3GAL1* beta-galactoside alpha-2,3-sialyltransferase 1, *THBD* thrombomodulin, *VUS* variant of unknown significance, *VWF* von Willebrand factor

### Case presentation

A 27-year-old male with no significant prior medical history presented with acute kidney injury (serum creatinine, 206 μmol/L), anemia (hemoglobin, 97 g/L), and thrombocytopenia (platelets, 96 × 10^9^/L) (Table 3). Renal replacement therapy (RRT) was initiated soon after initial presentation due to a rapid decline in kidney function, with a peak serum creatinine of 686 μmol/L. The patient received five plasma exchanges with improvement in his hematological parameters but not in his renal function, and he was started on eculizumab 900 mg every two weeks. After 56 months of treatment, he had sufficient renal recovery to discontinue RRT, with a new baseline serum creatinine of 144 μmol/L. No obvious potential triggers for this acute presentation were found.

**Table 3:** Laboratory investigations at initial presentation for the case study

Parameter	Value	Normal range
Urea	14.8 mmol/L	3.2–7.1 mmol/L
Creatinine	206 μmol/L	58–110 μmol/L
Estimated GFR	37 mL/min/1.73 m^2^	≥ 60 mL/min/1.73 m^2^
Albumin	26.6 g/L	33.0–55.0 g/L
Lactate dehydrogenase	2629 U/L	313–618 U/L
Bilirubin non-glucuronidated	15 μmol/L	0–19 μmol/L
Alkaline phosphatase	35 U/L	38–126 U/L
Alanine aminotransferase	25 U/L	≤ 49 U/L
C3	0.66 g/L	0.90–1.80 g/L
C4	0.21 g/L	0.15–0.53 g/L
Haptoglobin	< 0.10 g/L	0.30–2.00 g/L
CRP	< 5.1 mg/L	≤ 10.0 mg/L
ESR	25 mm/h	0–5 mm/h
INR	1.1	0.9–1.2
APTT	32 s	28–43 s
Leukocytes	7.9 × 10^9^/L	4.0–11.0 × 10^9^/L
Platelets	96 × 10^9^/L	150–400 × 10^9^/L
Erythrocytes	3.09 × 10^12^/L	4.40–6.00 × 10^12^/L
Hemoglobin; blood	97 g/L	135–175 g/L
Hematocrit; blood	0.268 L/L	0.400–0.540 L/L
Neutrophils; blood	5.8 × 10^9^/L	2.0–7.5 × 10^9^/L
Lymphocytes; blood	1.7 × 10^9^/L	1.2–3.5 × 10^9^/L
Monocytes; blood	0.2 × 10^9^/L	0.0–0.8 × 10^9^/L
Eosinophils; blood	0.1 × 10^9^/L	0.0–0.5 × 10^9^/L
Basophils; blood	0.1 × 10^9^/L	0.0–0.2 × 10^9^/L
Glucose	4.9 mmol/L	4.1–6.0 mmol/L

*APTT* activated partial prothromboplastin time, *C3* complement component 3, *C4* complement component 4, *ESR* erythrocyte sedimentation rate, *GFR* glomerular filtration rate, *INR* international normalized ratio

Upon genetic testing, two variants were identified. The first was a heterozygous variant, NM_172359.2 (c.191G > T;p.Cys64Phe) in *MCP/CD46*. While this variant has not been reported in population or clinical databases (gnomAD/ClinVar), it was previously reported in two unrelated patients diagnosed with aHUS [21, 22]. In silico predictions of the effect of this amino acid change on protein structure and function predicted a damaging effect. Based on the current literature, we classified this variant as a likely pathogenic variant (ACMG category 2).

The second variant was a heterozygous variant, NM_000204.4(*CFI*):c.1071 T > G, p.(lle357Met), detected in the *CFI* gene. This variant has been reported at low allele frequency in the population database (gnomAD: 0.0036%). In silico prediction of the effect of this amino acid change on protein structure and function was inconsistent. This variant has previously been reported in the homozygous state in two patients with aHUS [23], as well as in patients with FI deficiency [24–26]. Based on the current literature, this variant was classified as a likely pathogenic variant (ACMG category 2).

Following segregation analysis, it was found that this patient’s clinically asymptomatic mother (normal renal function, urine dipstick, and hematological profile) carried the *CFI* variant but not the *CD46* variant. This observation suggests that either (1) the *CFI* variant is not contributing to disease in this family, (2) the asymptomatic mother demonstrates incomplete penetrance of the *CFI* variant, or (3) this variant may have a synergic or additive effect with the second variant in the *CD46* gene in the proband. Unfortunately, paternal DNA was not available for further assessment. An asymptomatic sibling was clinically reviewed but, following genetic counseling and considering the difficulty in interpretation of genetic findings, the decision was made not to proceed with further testing at this time.

## Discussion

We have implemented an NGS panel test for the genetic evaluation of patients with a clinical or suspected diagnosis of aHUS and assessed its utility in the identification of clinically actionable genetic variants. To our knowledge, this is the first time molecular genetic testing was implemented in the clinical setting as a first-tier assessment, aimed at streamlining aHUS diagnosis. Using ACMG guidelines for variant classification and considering gene-disease validity, we identified only three patients (1.8%) with a confirmed clinical diagnosis; a total of four P/LP variants were identified in these three patients.

In contrast to our results, previous studies suggests that 30–60% of individuals with aHUS carry a variant in a gene of the complement system [8, 11]. One of the main differences between our study and previous studies is that our assay was used for initial diagnostic testing. Further, alternative causes of HUS, such as Shiga toxin, *S. pneumoniae*, or thrombotic thrombocytopenic purpura (TTP; ADAMTS 13 activity < 10%), were not tested or documented in most of the cases included in our study, nor was prior biochemical testing completed. We suspect that this has resulted in the observed dilution of the diagnostic yield, as the current cohort is more representative of individuals with all forms of HUS/complement-mediated TMA than confirmed cases of aHUS. To reflect real-life clinical practice, the indication for testing was determined by the primary referring physician rather than the testing laboratory, meaning results are reflective of the use of genetic testing in the clinical rather than research setting. Moreover, high-risk parameters for genetic disease, such as young age of onset and a positive family history, were not highly prevalent in our population and therefore may further account for the very low diagnostic yield obtained post-genetic testing. These findings raise important issues around the need for clear clinical guidelines on whom and when genetic testing should be performed in cases of suspected aHUS.

Further, most previous studies were case–control studies and did not always utilize ACMG guidelines for variant classification (with some published before guideline availability) and/or included *CFH*/*CFH*-related (CFHR) gene rearrangements and *CFH* polymorphisms as disease-associated variants [27]. A more recent study, using a cohort of individuals that met specific criteria for aHUS, showed the prevalence of P/LP variants in known causative genes to be approximately 14% [28]. Thus, we believe that the very low frequency of P/LP variants (1.8%) identified in our study may result from (1) use of more stringent variant classification criteria using ACMG guidelines, (2) less stringent criteria for selection of patients referred for genetic testing, and (3) inclusion of a cohort with lower risk features for genetic disease (predominately adult onset disease with no family history of disease), ultimately resulting in low diagnostic yield.

Eculizumab, a complement inhibitor, has shown excellent efficacy and safety in patients with aHUS; however, it is associated with high costs [29–31]. Identifying genetic variants in patients with aHUS is of clinical relevance, both to confirm diagnosis and to optimize patient management and treatment; however, the limitations of such testing must also be considered. Ideally, genetic testing should be incorporated into the diagnostic pathway—alongside clinical evaluation and other diagnostic tests, such as plasma complement factor concentrations, anti-factor H antibodies, and MCP expression—to help physicians more efficiently confirm diagnosis and inform treatment selection. However, genetic testing in patients with aHUS has several challenges, including the difficulty of genetic risk estimation and variant classification. Our case highlights the ongoing challenges of incomplete penetrance and the clinical implication for patients and families when performing variant interpretation of complement mediated genes.

Incomplete penetrance refers to the phenomenon where a phenotype is not expressed even though an individual carries the relevant genotype. Prior studies of family members of patients with aHUS have demonstrated incomplete penetrance across most (48–64%) complement-mediated genes; this remains an important parameter to consider in all individuals undergoing genetic testing [11, 32, 33]. The reasons for this are multifaceted: firstly, segregation analysis to help confirm pathogenicity of identified variants may be limited; and secondly, the precise risk of aHUS occurrence in family members presenting with the same variation as the affected proband is unclear. As illustrated by the presented case study, the proband diagnosed with aHUS had heterozygous variants in *CFI* and *CD46*; although the proband’s mother also carried the *CFI* variant, she was asymptomatic for aHUS, which may be attributed either to a lack of contribution of this variant to disease or to incomplete penetrance associated with this gene. Screening of additional family members may have contributed further data; however, following genetic counseling, further testing of asymptomatic family members was not pursued, meaning the exact contributions of *CFI* and *CD46* gene variants to aHUS disease pathogenesis remain ambiguous in this case. In addition, age at onset and severity of the disease may differ among family members with the same variation. Due to the above, genetic counseling is challenging and testing unaffected family members is not recommended. The low penetrance and variable expression of these genetic variants also complicate the interpretation of variants present in the population at higher frequencies and can limit the use of segregation and familial studies. In many cases, identified variants appear as risk factors for aHUS rather than its direct and unique cause, making classification according to ACMG guidelines challenging.

In contrast to the low rate of P/LP variants, we identified VUS in 25 (15%) patients referred for genetic testing, which may have clinical significance as more studies become available. Interpretation of these variants is described in Table 2. Most variants classified as VUS are rare variants with unknown functional or clinical evidence. We also assessed a selection of candidate genes, including genes with sporadic evidence linking them with aHUS, and variants with insufficient evidence for gene association with aHUS but which have been associated with other conditions. For example, loss of function variants in the *C9* gene are known to be associated with C9 deficiency; however, it is not known whether variants in *C9* contribute to the pathogenesis of aHUS [34]. *C9* and *C3* have been described as markers of complement activation in kidney biopsy specimens from patients with aHUS [35], but there is limited evidence of variants in *C9* causing aHUS, and the mechanism of disease, whether loss or gain of function, has not been well elucidated. Although we have reported two loss of function variants in *C9* (Arg116* and Arg194*), these variants were described as pathogenic variants for C9 deficiency but were of unknown significance for aHUS. A similar scenario is also observed for *VWF*. While pathologic mechanisms of aHUS caused by *VWF* variants have not been well described, multiple studies have reported the implications of VWF protein abnormalities in aHUS, including reduced amounts of high molecular weight VWF in the acute phase of the disease and the presence of large multimers in the plasma of relapsing patients [10, 36]. There seem to be multiple links between VWF and the alternative complement system, e.g., interactions of ultra large VWF with C3b to promote C3bBb assembly and interaction of VWF multimers with C3b to sustain tonic C3b inactivation by CFI [37]. Twenty-eight (16.8%) patients referred for genetic testing had an identified variant in a candidate gene. These cases highlight the ongoing need for further data on candidate genes, with routine review at regular intervals of gene panels to ensure the most up to date information is incorporated into clinical testing platforms.

It should be noted that *CFH* and *CFHR* gene rearrangements and deletions are not currently reported by our center. Such rearrangements have shown a high prevalence in aHUS and account for many of the positive genetic tests in previous studies. *CFHR1/CFHR3* deletions are common in the population (2–50%) [38, 39], with homozygous deletions associated with aHUS risk in the context of CFH autoantibodies [40]. Because of their frequency in the population, these are considered susceptibility variants and should not be included, in our opinion, in constitutional molecular testing. The hybrid *CFH*/*CFHR1* gene encodes a fusion protein and has been associated with aHUS risk and poor outcomes [35, 41]. This hybrid is reported in up to 5% of aHUS cases and has demonstrated less than 50% penetrance. The hybrid *CFH*/*CFHR1* gene has traditionally been identified by MLPA analysis of the deletion and/or duplication generated by this rearrangement [42]. However, CNV detection using our NGS pipeline is challenging considering sequence homology within *CFHR* genes and the high copy number variability within this region in the population, which makes interpretation of these common variants challenging. This hybrid has been shown to encode a protein product identical to a functionally significant *CFH* mutant (S1191L and V1197A), also observed as a *CFHR1* mutant (L290S and A296V) that has been previously described in association with aHUS [41, 43]. Detection of these variants could possibly highlight the presence of the hybrid gene in our NGS pipeline [43]. Technologies other than NGS, including MLPA, comparative genomic hybridization array, and long-range polymerase chain reaction, could be performed in parallel to identify these complex rearrangements. However, their clinical actionability in a constitutional setting remains unclear.

Lastly, while additional antigenic, biochemical, and functional tests to screen for abnormalities in the alternative complement system may provide valuable information, assisting both in the clinical interpretation of genetic variants and the diagnosis of aHUS, the results of such tests were unfortunately not provided for the cases referred to our center. We acknowledge that it is important for physicians to combine all available and relevant data to establish a final diagnosis of aHUS.

## Conclusions

This study describes the first 2.5 years’ experience of using clinical NGS panel testing for the diagnosis of aHUS. We observed a very low prevalence of clinically significant variants compared with previous studies and our findings highlight the challenges in variant classification, gene–disease association, and copy number variant detection and interpretation. To increase the utility of NGS panel testing for aHUS diagnosis, we propose that accurate and specific genetic testing should be interpreted alongside biochemical and clinical information for patients with suspected disease, in order to provide rapid and tailored disease diagnosis and management, which should lead to better patient outcomes. These findings also emphasize the need for a more tailored approach to patient referral for genetic testing, such as incorporation of specific inclusion (i.e., positive family history and clinical indication) and exclusion (i.e., secondary causes of aHUS) criteria.

## Supplementary Information

Below is the link to the electronic supplementary material.

Supplementary file1 (DOCX 59 KB)

## Data Availability

The original contributions presented are included in the manuscript; further inquiries should be directed to the corresponding author.
