# Supplementary material for: Genotypic analysis of a large cohort of patients with suspected atypical hemolytic uremic syndrome
Source: J Mol Med (Berl). 2023 Jul 19;101(8):1029–40. doi: 10.1007/s00109-023-02341-4 (PMC10400659; doi:10.1007/s00109-023-02341-4)
Supplement: Supplementary file 1 — Supplementary file1 (DOCX 59 KB) [file 109_2023_2341_MOESM1_ESM.docx]

**Supplementary Table 1** List of genes tested in the NGS panel

| **Gene** | **Accession number** | **Minimum coverage – average (SD)** | **Average coverage – average (SD)** |
| --- | --- | --- | --- |
| ***C3*** | NM_000064.2 | 1064 (473) | 1395 (617) |
| ***C9*** | NM_001737.4 | 979 (501) | 1303 (642) |
| ***CD46*** | NM_172359.2 | 962 (509) | 1268 (660) |
|  | NM_002389.4 | 1086 (696) | 1341 (797) |
| ***CFB*** | NM_001710.5 | 1143 (550) | 1453 (686) |
| ***CFH**** | NM_000186.3 | 963 (531) | 1416 (758) |
|  | NM_001014975.2 | 1071 (528) | 1134 (555) |
| ***CFHR1**** | NM_002113.2 | 1013 (696) | 1530 (1014) |
| ***CFHR2*** | NM_005666.3 | 1193 (649) | 1666 (926) |
| ***CFHR3**** | NM_021023.5 | 848 (656) | 1251 (868) |
| ***CFHR4**** | NM_001201550.2 | 1058 (609) | 1528 (853) |
| ***CFHR5*** | NM_030787.3 | 940 (502) | 1403 (722) |
| ***CFI*** | NM_000204.3 | 943 (510) | 1282 (660) |
| ***DGKE*** | NM_003647.2 | 885 (449) | 1210 (594) |
| ***F12*** | NM_000505.3 | 1105 (516) | 1439 (656) |
| ***FKRP*** | NM_001039885.2 | 775 (360) | 1331 (600) |
| ***INF2**** | NM_022489.3 | 1031 (478) | 1389 (641) |
|  | NM_001031714.3 | 1448 (867) | 1629 (976) |
| ***MMACHC*** | NM_015506.2 | 978 (459) | 1316 (626) |
| ***MMADHC*** | NM_015702.2 | 975 (531) | 1298 (685) |
| ***PLG*** | NM_000301.3 | 998 (510) | 1331 (650) |
| ***ST3GAL1*** | NM_003033.3 | 1176 (538) | 1572 (712) |
| ***THBD*** | NM_000361.2 | 831 (327) | 1525 (714) |
| ***VWF**** | NM_000552.3 | 1061 (509) | 1431 (675) |

*Due to sequence complexity, the following regions were not assessed for copy number variants: *CFHx20*, *CFHR1x04*, *CFHR3x05*, *CFHR4x09*, *VWFx28*, and *INF2x08*.

C3, complement component 3; C9, complement component 9; CD46, cluster of differentiation 46; CFB, complement factor B; CFH, complement factor H; CFHR, complement factor H-related; CFI, complement factor I; DGKE, diacylglycerol kinase epsilon; F12, coagulation factor XII; FKRP, fukutin-related protein; INF2, inverted formin-2; MMACHC, metabolism of cobalamin associated C; MMADHC, metabolism of cobalamin associated D; PLG, plasminogen; SD, standard deviation; ST3GAL1, beta-galactoside alpha-2,3-sialyltransferase 1; THBD, thrombomodulin; VWF, von Willebrand factor.
